# Prognostic performance of MR-pro-adrenomedullin in patients with community acquired pneumonia in the Emergency Department compared to clinical severity scores PSI and CURB

**DOI:** 10.1371/journal.pone.0187702

**Published:** 2017-11-21

**Authors:** Jacopo Maria Legramante, Maria Mastropasqua, Beniamino Susi, Ottavia Porzio, Marta Mazza, Grazia Miranda Agrippino, Cartesio D′Agostini, Antonella Brandi, Germano Giovagnoli, Vito Nicola Di Lecce, Sergio Bernardini, Marilena Minieri

**Affiliations:** 1 Emergency Department, Policlinico Tor Vergata, Roma, Italy; 2 Department of Medical Systems, Università di Tor Vergata, Roma, Italy; 3 Clinical Laboratory, Ospedale Pediatrico Bambino Gesù, IRCCS, Roma, Italy; 4 Department of Experimental Medicine and Surgery, Università di Tor Vergata, Roma, Italy; 5 Medicina e Chirurgia d’Accettazione e d’Urgenza, Ospedale Maria Vittoria, Torino, Italy; 6 Medicina per l’Alta Complessità Assistenziale 3, AOU Careggi, Firenze, Italy; 7 Laboratory of Clinical Microbiology, Policlinico Tor Vergata, Roma, Italy; 8 Laboratory of Clinical Biochemistry, Department of Laboratory Medicine, Policlinico Tor Vergata, Roma, Italy; The University of Tokyo, JAPAN

## Abstract

**Aim:**

(i) evaluate the performance of MR-pro-ADM in reflecting the outcome and risk for CAP patients in the emergency department, and (ii) compare the prognostic performance of MR-pro-ADM with that of clinical scores PSI and CURB65.

**Methods:**

Observational prospective, single-center study in patients with suspected community acquired pneumonia (CAP). Eighty one patients underwent full clinical and laboratory assessment as by protocol, and were followed up a 28 days. Primary endpoints measured were: death, death at 14 days, non-invasive mechanical ventilation (NIMV), endotracheal intubation (EI), ICU admission, overall hospital stay >10 days, emergency department stay >4 days. The discriminative performance of MR-pro-ADM and clinical scores was assessed by AUROC analysis.

**Results:**

The distribution for MR-pro-ADM followed an upward trend, increasing with the increase of both PSI (p<0.001) and CURB65 (p<0.001) classes. However, the difference between MRproADM values and score classes was significant only in the case of CURB65 classes 0 and 1 (p = 0.046), 2 (p = 0.013), and 3 (p = 0.011); and with PSI classes 5, 3 (p = 0.044), and 1 (p = 0.020).

As to the differences among variables for the six end-points, MR-pro-ADM values in the two groups selected for each considered end-point differed in a statistically significant manner for all endpoints. Both PSI and CURB65 differed significantly for all end-points, except for stay in the ED longer than 4 days and the hospital stay longer than 10 days and endotracheal intubation (only PSI classes differed with statistical significance).

ROC analyses evidenced that MR-pro-ADM values gave the greatest AUC for the prediction of death, endotracheal intubation, hospital stay >10 days and DE stay >4 days, compared to the PSI and CURB (though difference not statistically significant).

For each endpoint measured, the best thresholds values for Mr-pro-ADM were: 1.6 (specificity 76.5%; sensitivity 77.8%) for death; 2.5 (specificity 88.9%; sensitivity 80.0%) for death at 14 days; 1.5 (specificity 77.0%; sensitivity 87.5%) for NIMV; 2.4 (specificity 88.7%; sensitivity 83.3%) for endotracheal intubation; 0.9 (specificity 53.5%; sensitivity 70.6%) for DE stay greater than 4 days; 1.9 (specificity 82.1%; sensitivity 55.3%) for hospital stay greater than 10 days.

The AUC for the combination of MR-pro-ADM and PSI was 81.29% [63.41%–99.17%], but not in a statistically significant manner compared to the AUCs of the single predictors. Conversely, the AUC for the combination of MR-pro-ADM and CURB65 was 87.58% [75.54%–99.62%], which was significantly greater than the AUC of CURB65 (p = 0.047) or PSI (p = 0.017) alone.

**Conclusions:**

The present study confirms that assessment of MR-pro-ADM levels in CAP patients in addition to CURB scores increases the prognostic accuracy of CURB alone and may help rule out discrepancies arising from flawed clinical severity classification.

With particular reference to patients scoring in the upper classes of CURB and PSI, MR-pro-ADM values provided additional information towards a better risk stratification of those patients. In particular, our results pointed towards two MR-pro-ADM threshold values that appear to predict with a good degree of accuracy the patient's need for non-invasive mechanical ventilation, endotracheal intubation, or intensive care. This aspect, however, deserves further investigation.

## Background

Among the infectious diseases in industrialized countries, Community Acquired Pneumonia (CAP) represents the most frequent cause of death [1], especially among elderly patients presenting several comorbidities [2]. The overall mortality rates for CAP range from 7.2% [2,3] up to 29% among ICU patients [2; 4,5].

The prognostic assessment of CAP includes several clinical severity scores plus several biochemical markers, each of which integrate and support the clinical evaluation with different information on the host’s inflammatory response (i.e., the main determinant factor of clinical evolution [6]) and biochemical/physiological aspects.

Two widespread clinical scores in use today are the pneumonia severity index (PSI) and CURB-65 [7,8]. The PSI is considered the reference standard for classifying severity and determining 30-day mortality risk of pneumonia patients by means of a two-step algorithm based on a 20-item scoring system considering age, comorbidities, physical examination and laboratory parameters. Initially validated as a prognostic tool on data from over 50.000 patients it is also employed for discriminating CAP patients with a low mortality risk suitable for home management from those with low risk but requiring hospitalization; however, its computation method tends to allocate an excessive weight to age and comorbidity factors, classifying patients in higher risk classes (IV and V); additionally it has a 10% intra-observer variation and a poor predictive value for mortality [9,10]. The CURB-65 (confusion, urea, respiratory rate, blood pressure, age >65) score has been proposed as a simpler alternative; it is specifically predictive for mortality [11], but features moderate sensitivity and specificity. It is generally accepted that a PSI>4 and CURB65>3 are indicative of severe illness.

As for biochemical markers, those most currently in use are CRP and PCT, in virtue of their high predictive capacity [10]. Specifically, PCT has been extensively studied in several setting-specific and disease-specific populations (such as ED, Intermediate care units, healthy individuals, patients with LRTI and others) [12–15] and has been shown to be a highly reliable marker for disease severity, disease progression, guiding antibiotic therapy, and identifying patients at need of intensive care. In the setting of CAP, however, the prognostic accuracy of PCT concentration is low as it yields overlapping values for different disease severity [10] and only small differences between survivors and non-survivors.

Recently new attention is being focused on adreno-medullin and specifically on its more stable midregional fragment pro-adrenomedullin (MR-pro-ADM) [16], which has demonstrated to be the most reliable biomarker available in mortality and prognosis prediction for pneumonia (and many other diseases) [17–21]. Adrenomedullin is a potent vaso-dilating, antimicrobial, immunomodulant peptide [22–24]. Being a hormokine belonging to the Calc gene family, it is synthetized by endocrine cells [25–27] and secreted systemically from various cell types, involved in the inflammatory response as a result of stimulation by various factors during sepsis, such as catecholamines, hypoxia, oxidative stress, inflammatory mediators, and cytokines. Most of these factors are originated from the acute pathophysiological changes of sepsis and are not associated with comorbidities [10, 27]. Pro-adrenomedullin measured upon admission has demonstrated to be a good predictor of severity and outcome of CAP and can improve the prognostic accuracy of CURB65 used alone [10; 27].

To date, little evidence has been collected on the use of MR-pro-ADM in the acute setting of the Emergency Department (ED). Therefore, the present study aims to: (i) to evaluate the possible role of MR-pro-ADM in reflecting the outcome and risk for patients affected by CAP in the emergency department, and (ii) to compare the prognostic value of this biomarker with that of the most widely used clinical scores in CAP, namely, PSI and CURB65.

## Materials and methods

The present work was an observational, prospective, single-centre study within the Complex Operative Unit of Emergency Medicine of the Policlinico Tor Vergata (Rome, Italy) and enrolled 81 patients referred to our Emergency Department between December 2012 and March 2015. The study protocol was approved by the Ethics Committee of the Policlinico Tor Vergata (Rome) and was performed in accordance with the principles set out in the Declaration of Helsinki; all patients provided written informed consent prior to inclusion.

Inclusion criteria were: age ≥18 years, suspected diagnosis of community-acquired pneumonia as defined by the Infectious Disease Society of America/American Thoracic Society guidelines [28], new clinical symptoms of lower respiratory tract infection (fever, cough, sputum, chest objectivity) and new radiological findings of lower respiratory tract infection (LRTI). Exclusion criteria were: admittance to hospital in the last 10 days, pneumonia in the previous 30 days; being affected by nosocomial pneumonia, cystic fibrosis, active tuberculosis; immuno-deficiency (due to HIV infection, neutropenia with neutrophil count <1000/mm3, previous transplantation, immunosuppressive therapy, hematological neoplasm); and pregnancy.

Upon admission to the emergency department (ED), the patient's demographic data and clinical history were collected. Patients then underwent assessment of clinical (blood pressure, heart rate, respiratory rate, oxygen saturation, body temperature) and laboratory parameters (full blood cell count, C-reactive protein, procalcitonin, renal function, and electrolytes), and arterial blood gas analysis. Their clinical severity was also determined based on the Kelly-Matthey scale [29]. The Chest X-rays or CT scans were performed depending on the ED physician's clinical assessment and were further reviewed by the emergency department radiologist. When necessary, analyses of blood culture, sputum, urine, bronchial aspirate or bronchoalveolar samples were also performed. Patient CURB65 and PSI were calculated according to international criteria. The final diagnosis was considered the one provided by the ED physician on shift at the time of discharge. Patients were followed-up (in the ED and/or in ward) at 14 and 28 days for data on need for non-invasive and/or invasive mechanical ventilation (NIMV, EI), death or ICU admission, and length of hospital stay.

MR-pro-ADM was detected in EDTA-plasma from blood obtained from peripheral venous catheters, as soon as possible after the informed consent was obtained. The sample was stored at– 20°C and analyzed, at a later stage, by a time-resolved amplified cryptate emission technology assay (TRACE—Kryptor MR-pro-ADM; BRAHMS AG, Hennigsdorf, Germany). The MR-pro-ADM assay has a measuring range from 0 to 100 nmol/L; the limit of detection and limit of quantification were 0.05 and 0.23 nmol/L, respectively; the intra assay coefficient of variability (CV) was 1.9% and the inter laboratory CV was 9.8%. The physician was blinded about the results of biomarker value.

### Primary endpoints

The primary endpoints of interest were death, death at 14 days, non-invasive mechanical ventilation, endotracheal intubation, length of stay in the ED and overall hospital length of stay (LOS). Two of the primary endpoints, length of stay in the ED and overall hospital length of stay, were dichotomized using their median as threshold value thus creating the variables, ED4 (length of stay in the Emergency Department> 4) and TDH10 (overall hospital length of stay > 10), thus having a total o 6 endpoints.

## Statistical analyses

Distributions of variables were assessed with Kolmogorov-Smirnov non-parametric test. According to their distributions, variables were expressed as mean ± SD, median and interquartile range, or proportions. Analogously, between-sample comparisons were performed with unpaired samples t-test, Mann-Whitney non-parametric test or χ2-test with Yate’s correction.

The distribution for MR-pro-ADM in the groups characterized by the values taken by CURB65 and PSI was assessed with the Kruskal-Wallis non-parametric test (corresponding to the one-way ANOVA for normally distributed variables). For each of the trends observed we evaluated the statistical significance of the differences between each pair of groups. For this purpose we used a post-hoc Bonferroni adjusted Mann-Whitney test.

The capacity of CURB65, PSI and MR-pro-ADM in discriminating endpoints was assessed by ROC curves. For each of the three predictors, AUC values were reported with a 95% CI. The optimal threshold for MR-pro-ADM and specificity and sensibility were reported. The comparison between the three AUCs was performed with the non-parametric test of DeLong [30].

For the death endpoint, the incremental predictive improvement of MR-pro-ADM over PSI and CURB65 was assessed.

For all the statistic tests, we considered as significant a p value <0.05.

## Results

### General patient characteristics

The final study population consisted of 77 patients (4 patients had been excluded due to incomplete information). Baseline characteristics of the study population are summarized in Table 1. The patient population had a mean age of 69.6 ± 17.4 years; 62 patients (80.5%) had at least one comorbidity.

**Table 1:** Patient demographic and clinical parameters.

	Overall (n = 77)
Male Gender (n)	47 (61.0%)
Age (yrs)	69.57 ± 17.43
*Comorbidities (n)(%)*	
Congestive heart failure	32 (41.6%)
Kidney failure	21 (27.3%)
Liver disease	4 (5.2%)
COPD	37 (48.1%)
Tumor	3 (3.9%)
Diabetes	12 (15.6%)
Encephalopathy	23 (29.9%)
*Hospitalization*	
Discharge without hospitalization (n)	19 (24.7%)
Hospitalization (n)	58 (75.32%)
Hospitalization in ED (days)	4.4 ±2.96
Total hospitalization (days)	10 [7–15]
ICU admission	9 (11.7%)
Deaths at 14 days (n)	5 (6.5%)
Death (n)	9 (11.7%)
*Respiratory interventions*	
Non-Invasive Mechanical Ventilation (n)	16 (20.78%)
Endotracheal intubation (n)	6 (7.8%)
*Clinical parameters at admission*	
Systolic pressure (mmHg)	134 ± 23.7
Diastolic pressure (mmHg)	74 ± 13.8
Heart rate (bpm)	100 ± 21.85
Ventilation rate (brpm)	20.04 ± 5.53
Oxygen saturation	91 ± 11.5
Temperature	37 ± 1.03
*Laboratory parameters*	
ph	7.39 ± 0.11
White cells (n)	12.24 ± 5.23
Blood gas (PaCO_2_)	58 [50–75]
CRP (mg/L)	83.4 [19.09–135.75]
MRproADM (nmol/L)	1 [0.55–1.76]
*Clinical severity scores*	
CURB65	2 [1–2]
PSI	4 [2–5]
Kelly-Matthay	1 [1–2]

While clinical scores were obtained for all patients, PCT had been measured (as needed) on 65 patients (84.4%), showing a value of 0.23 [0.13–0.59]. The value of MR-pro-ADM on the whole sample was 1 [0.55–1.76] nmol/L.

The primary endpoints of interest were: death (n = 9, 11.7%); death at 14 days (n = 5, 6.5%); non-invasive mechanical ventilation (NIMV) (n = 16, 20.8%); endotracheal intubation (EI) (n = 6, 7.8%); ICU admission (n = 9, 11.7%); overall hospital stay greater than 10 days (n = 38, 49%); ED stay greater than 4 days (n = 34, 44%).

As to the relationship between MR-pro-ADM and gender, MR-pro-ADM value was 1.32 [0.74–2.43] 1 nmol/L in men, and 0.90 [0.46–1.53] in women; the difference was not statistically significant (p = 0.064), though it showed a slight upward trend as age increased (Supplementary material S1 Fig), in accordance with data in literature [31].

Scores of clinical severity *PSI and CURB65* provided inconsistent classification of patients: PSI scores set 60% of patients in classes IV and V (median age 79.2 ± 8.2), while 55% of the remaining patients were in class 1, with 83,4% being under 70 years of age (median age 43.2 ± 12.6) and having no comorbidities. Conversely, CURB65 score placed approximately half of the patients (49.4%) in class 0–1, and 24% in class 3.

**Fig 1:**
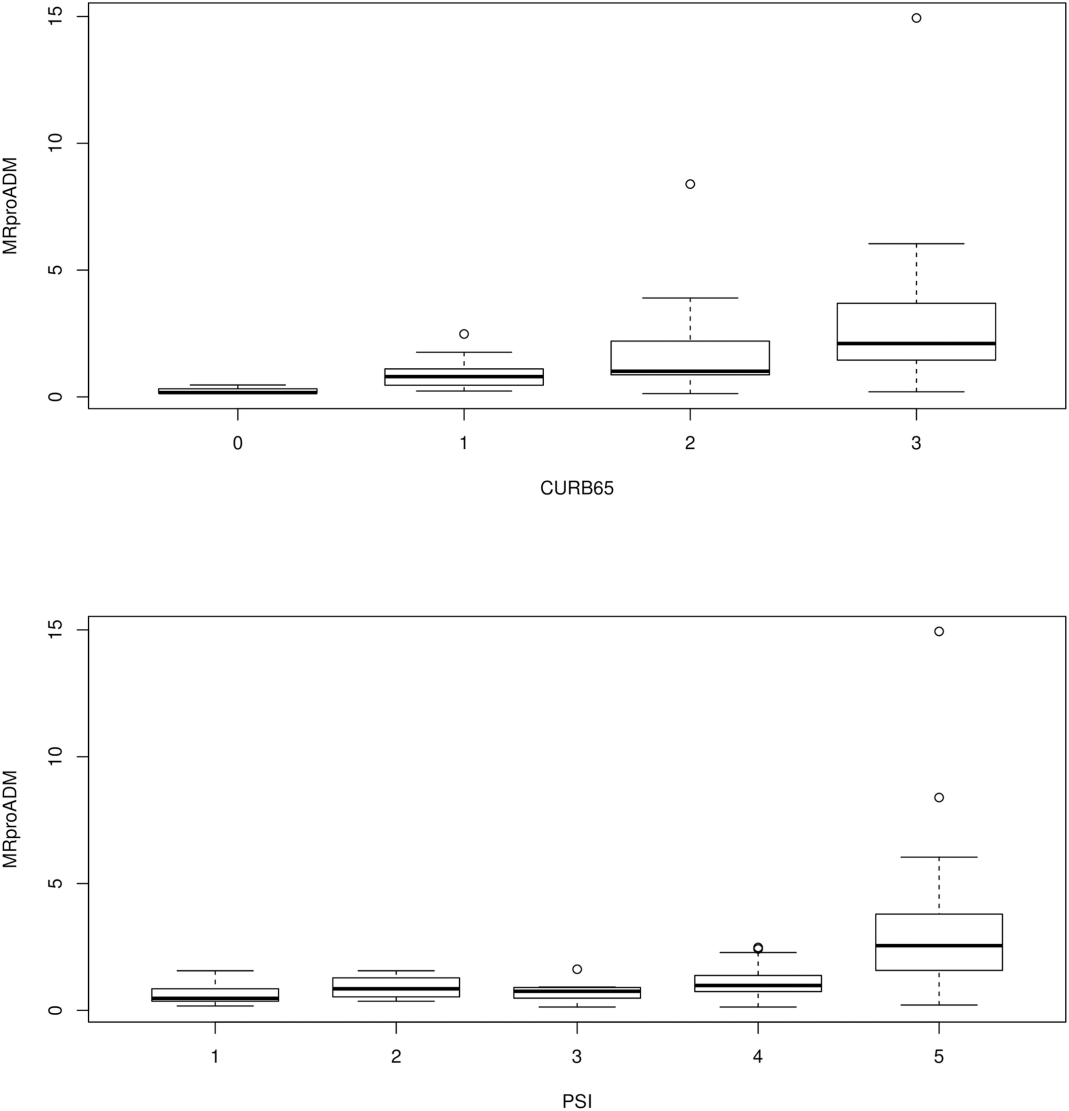
The distribution for MR-pro-ADM values within classes of clinical severity scores. Upper panel: MR-pro-ADM distribution within CURB65 classes: statistically significant differences in MR-pro-AMD concentrations were found between CURB65 group 0 and 1 (p = 0.046); between CURB65 group 0 and 2 (p = 0.013); and between CURB65 group 0 and 3 (p = 0.0108). Lower panel; MR-pro-ADM distribution within classes PSI: statistically significant differences in MR-pro-AMD concentrations were found between PSI class 5 and 1 (p = 0.020), and between PSI class 5 and 3 (p = 0.044).

MR-pro-ADM, PSI and CURB65. The distribution for MR-pro-ADM (Fig 1) showed a significant trend within PSI (p<0.001) and CURB65 (p<0.001) classes, increasing with the increase of the two score classes, i.e. the higher the risk class, the higher MR-pro-ADM values. However, the differences between MR-pro-ADM values among score classes results significant only between CURB65 class 0 and classes 1 (p = 0.046), 2 (p = 0.013) and 3 (p = 0.011); and in the case of PSI between class 5 and classes 3 (p = 0.044) and 5 and 1 (p = 0.020).

The differences among variables for the six end-points (NIMV, EI, death, death at 14 days, hospital stay longer than 10 days, DE stay longer than 4 days) are reported in supplementary material (S1–S6 Tables). MR-pro-ADM values showed statistically significant differences in the two groups selected for each end-point considered (S1–S6 Tables). Both PSI and CURB65 differed significantly for all end-points, except for stay in the ED longer than 4 days (in which case neither were significant) and the hospital stay longer than 10 days and EI (only PSI classes differ with statistical significance).

**Fig 2:**
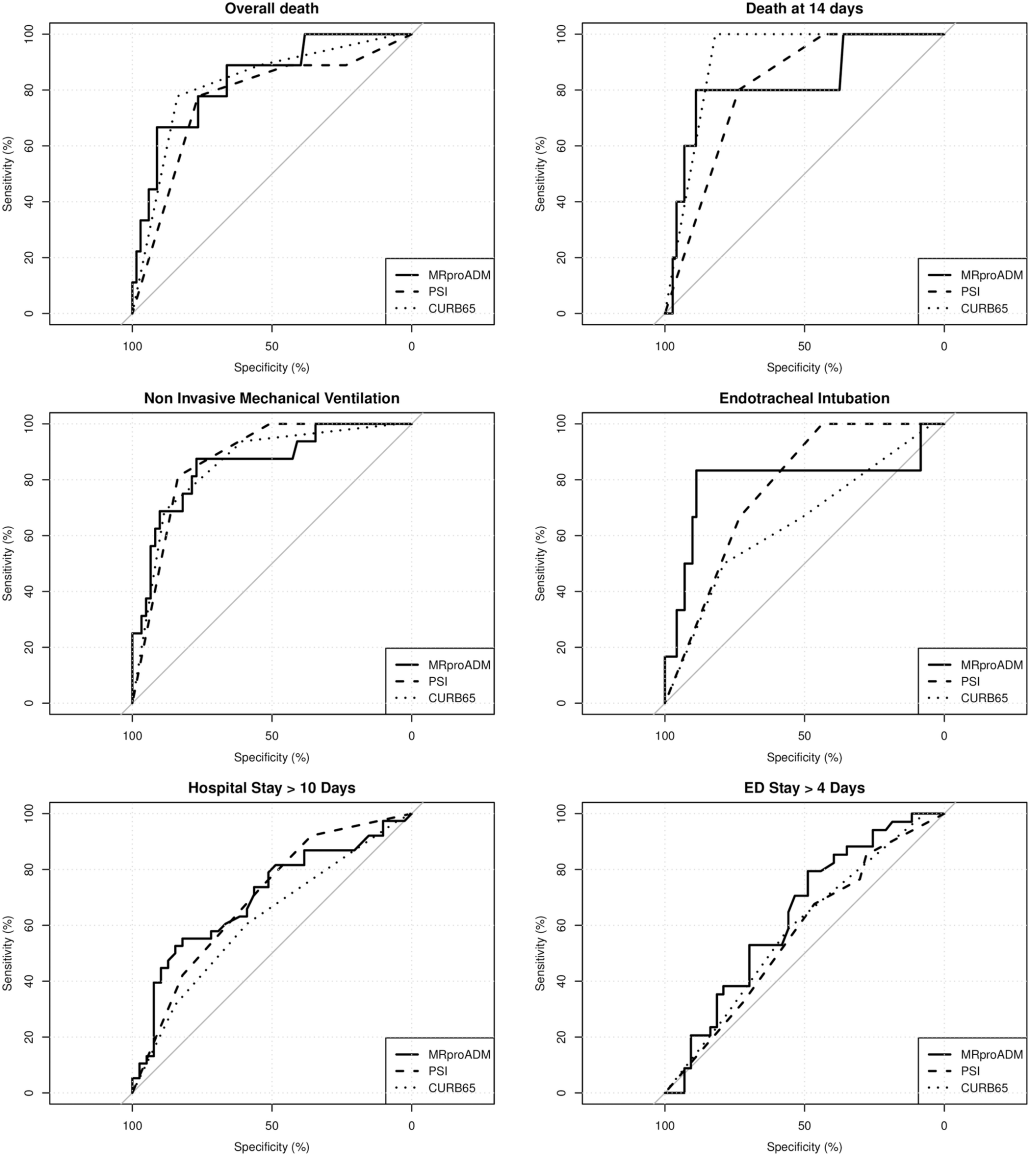
MR-pro-ADM prediction in relation to study outcomes: AUC with a 95% CI for each predictor MR-pro-AMD, CURB65 and PSI, by main endpoint. From upper left panel to lower right panel: overall death (CURB65: AUC [95% CI] = 0.824 [0.685–0.964], PSI: AUC [95% CI] = 0.766 [0.598–0.934], MRproADM: AUC [95% CI] = 0.837 [0.704–0.970]), death at 14 days (CURB65 –AUC [95% CI] = 0.910 [0.865–0.954], PSI:AUC [95% CI] = 0.811 [0.696–0.926], MRproADM: AUC [95% CI] = 0.824 [0.617–0.998]), non-invasive mechanical ventilation CURB65 –AUC [95% CI] = 0.855 [0.762–0.948], PSI: AUC [95% CI] = 0.872 [0.801–0.943], MRproADM: AUC [95% CI] = 0.858 [0.750–0.960]), endotracheal intubation (CURB65:AUC [95% CI] = 0.647 [0.418–0.875], PSI: AUC [95% CI] = 0.772 [0.648–0.896], MRproADM: AUC [95% CI] = 0.793[0.535–0.997]), total hospital stay>10 days (CURB65: AUC [95% CI] = 0.618 [0.502–0.735], PSI: AUC [95% CI] = 0.695 [0.582–0.807], MRproADM—AUC [95% CI] = 0.698 [0.580–0.817]), ED stay over 4 days (CURB65: AUC [95% CI] = 0.584 [0.466–0.702], PSI:AUC [95% CI] = 0.568 [0.444–0.691], MRproADM: AUC [95% CI] = 0.635 [0.512–0.758].

According to ROC analyses (Fig 2), MR-pro-ADM showed the greatest AUC for the prediction of death, endotracheal intubation, hospital stay longer than 10 days, and Emergency Department stay greater than 4 days. In all cases, none of the observed differences was found to be statistically significant. The best thresholds of MR-pro-ADM were: 1.6 (specificity 76.5%; sensitivity 77.8%) for death; 2.5 (specificity 88.9%; sensitivity 80.0%) for death at 14 days; 1.5 (specificity 77.0%; sensitivity 87.5%) for NIMV; 2.4 (specificity 88.7%; sensitivity 83.3%) for endotracheal intubation; 0.9 (specificity 53.5%; sensitivity 70.6%) for ED stay greater than 4 days; 1.9 (specificity 82.1%; sensitivity 55.3%) for hospital stay greater than 10 days.

**Fig 3:**
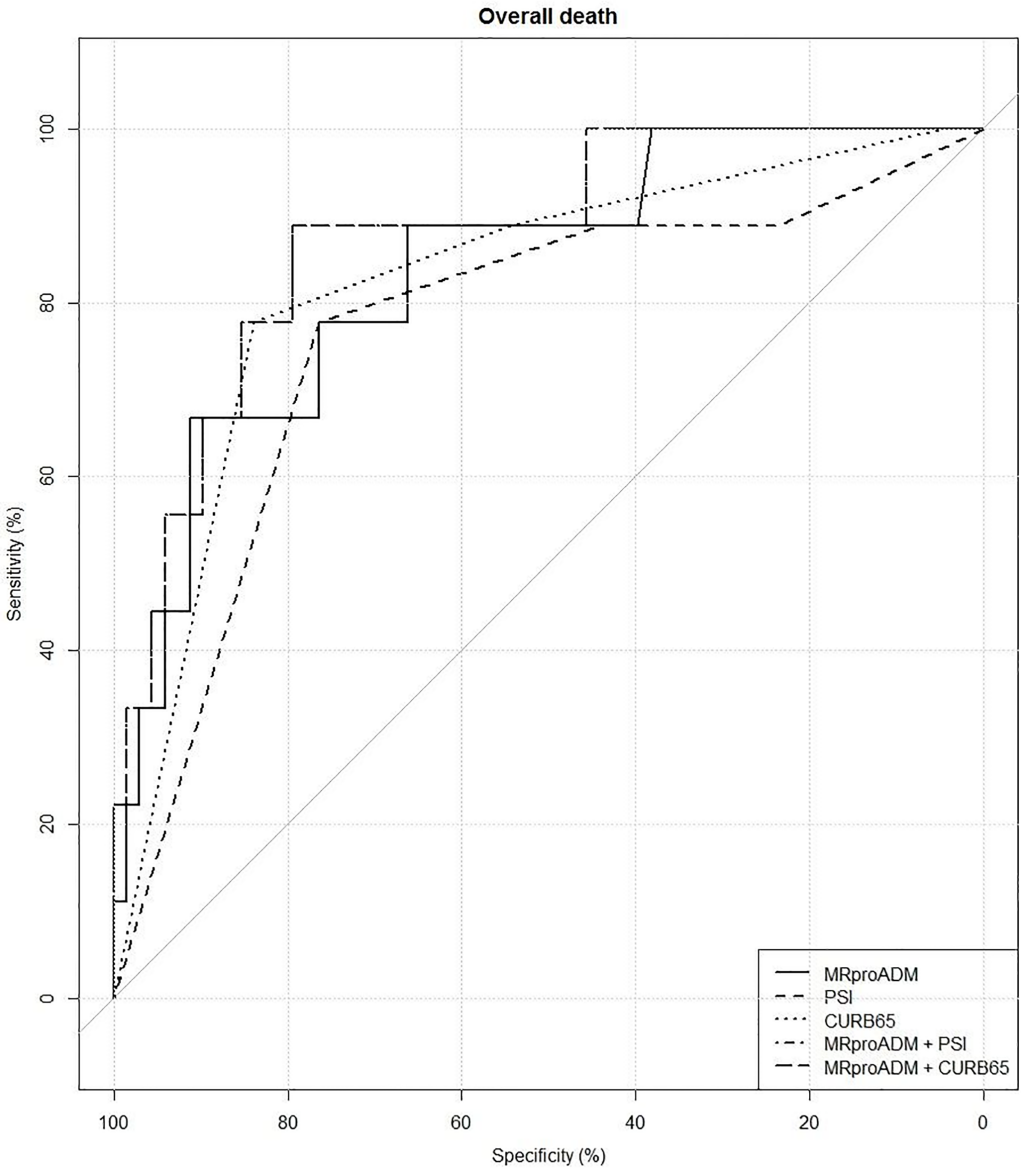
Predictive performance of MR-pro-ADM in combination with PSI or CURB65 for overall death.

To assess the predictive performance of the combination of MR-pro-ADM with PSI or CURB65, two multivariate logistic regression models were fitted to the death endpoint. The resulting ROC curves are reported in Fig 3. The AUC for the combination of MR-pro-ADM and PSI was 81.29% [63.4%–99.2%]; it wasn't found to be significantly different from the AUCs of the single predictors. The AUC for the combination of MR-pro-ADM and CURB65 was 87.6% [75.54%–99.62%]; it was significantly greater than the AUC of both CURB65 (p = 0.047) and PSI (p = 0.017) alone.

## Discussion

CAP is a leading cause of sepsis and the accurate estimation of disease severity is crucial for guiding therapeutic decisions and providing best possible care available to CAP patients. While less severe CAP cases can be managed at the patient’s home through appropriate home-based treatment, up to 30% of pneumonia patients require hospitalization, and approximately 20% require intensive care [32,33]. However, given the complexity of the disease, there is a general tendency to a cautious approach in the patient risk assessment with a consequent overuse of specialized hospital care and occupation of vital hospital resources that could be allocated elsewhere. The combined use of clinical severity scores and biomarkers in support to physician assessment are extremely useful [9, 27,34], but their predictive accuracy is still far from optimal [3,35], therefore to date, the identification of reliable markers for accurate prognosis and predictors of mortality is an open issue. The present study aimed to assess the prognostic accuracy of MR-pro-adrenomedullin (MR-pro-AMD), which has been shown to be a good predictive marker of severity and outcome for CAP, with a comparable accuracy of PSI, and that improves the prognostic accuracy of CURB65 used alone [31,34,36].

Our sample included elderly people, with many comorbidities, but with low severe pneumonia profile as described by clinical scores (77% CURB65 0–2, despite 60% were PSI IV-V). In accordance with literature, PSI did not show to be a good predictor for mortality as the score system tended to place many patients in higher classes because of age and comorbidities, whereas CURB65 seemed to better describe our sample, correlating more closely to mortality (p<0,01). It is possible that the predictive power of both PSI and CURB65 is limited to pneumonia *per se* not taking into account problems derived by comorbidities especially in elderly patients. Despite the low absolute number of deaths observed, most of them fell into CURB65 class 3 of and in PSI class 5. It is also noteworthy to mention that no universally accepted criteria have been proposed for severe CAP requiring admission to an ICU, thus making it difficult for emergency physicians to perform a risk stratification in these patients. MR-pro-ADM seems to have the potential to improve prognostic prediction of PSI and/or CURB65 in patients affected by CAP.

Indeed, our data demonstrate that MR-pro-ADM levels were linearly related to PSI and CURB65 score classes with a *p* value <0.01, thus confirming that higher values of MR-pro-ADM corresponded to higher PSI and CURB65 scores. Courtais [37] and Huang [38] reported narrow-ranged MR-pro-ADM values in lower PSI classes, while wide-ranged values in the highest score classes. The same event occurs when considering high classes of CURB65 [30]. The authors report that adrenomedullin may improve risk assessment in patients with high PSI score (4 and 5 classes), identifying a very high-risk subcategory. In line with this previous study, our results show that MR-pro-ADM values found in PSI classes 1 and 2 patients fell within a very narrow range (between 0.17 and 1.56 nmol/L, with an average of 0.69 ± 0.42 SD) as compared to those falling within classes 4 and 5, which showed wider variability, with a range from 0.13 to 8.39 and a mean value of 2.26 ± 2.55 SD even after having discarded outlier values.

This wider variability allows to identify lower values, with relatively good prognostic significance, from higher values with negative predictive values. Accordingly, the mortality rate in PSI classes 4 and 5 is 17,4%, however increasing up to 32% when considering the patients who have MR-pro-ADM value ≥1.6 nmol/L (mortality cut-off value, according to ROC curves) and decreasing to 4.2% when considering patients with value <1,6 mmol/L. Furthermore, mortality rate of CURB65 class 3 is 38.9% and this value increases to 45.5% for patients out of cut-off. Even considering the relatively small sample of our study, as demonstrated by the low number of deaths, we might conclude based on our data that adrenomedullin could be useful as an additional assay for sub-stratifying the upper classes of PSI and CURB65.

In our study we found that adrenomedullin levels at ED admission were much higher in patients who progressed toward severe sepsis or septic shock, showing a similar predictive significance as compared to PSI and CURB65, thus representing an additional and easy-to-determine prognostic tool.

Moreover, MR-pro-ADM correlates both with mortality (p<0.01) and complications of CAP, defined by the need for NIMV (p<0.01), EI (p<0.05) and ICU admission (p<0.05). Moreover, we shall remind that the pro-hormone levels correlate with all the end points considered, regardless of the statistical test (this does not occur for clinical scores). In line with our data, Espana et al [39] have shown that MR-pro-ADM evaluation adds a prognostic value to the clinical scores for both predicting complications and for risk stratification, identifying severe patients who need ICU admission. ROC curves analysis shows the possibility to define two threshold values: a lower one (1.6 nmol/L) for predicting mortality and NIMV, and a higher one (2.5 nmol/L) for predicting EI and intensive care unit admission. We suggest these threshold values as the values obtained for predicting mortality (1.6 nmol/L) and NIMV (1.5 nmol/L) are similar, as well as the values detected for predicting EI (2.4 nmol/L) and intensive care unit admission (2.6 nmol/L).

These findings confirm that MR-pro-ADM is a prognostic tool with a accuracy comparable to clinical scores and suggest that increasing the specificity of selection, we can identify patients truly requiring intensive care. The result provided by the random forest analysis further confirms the importance of MR-pro-ADM in the definition of risk for the patients with CAP: the peptide is one of the key points, along with the CURB65, since in its absence the accuracy of the prediction of mortality is greatly reduced.

This would allow patients truly requiring intensive care to be identified early in the Emergency Department, thus saving time and allocating the resources to those patients who would most benefit from them. We suggest patients whose MR-pro-ADM exceed the cut-off value (in particular 2.5 nmol/l) receive a more intensive monitoring and foresee, already upon presentation, non-invasive mechanical ventilation for those patients with MR-pro-ADM over 1.6 nmol/l. The specific threshold value though would need to be confirmed through further studies. This would allow an early risk stratification in a setting like Emergency Department where time and definition of appropriate level of care are “vital”.

Finally, further studies would be needed to investigate also any possible stratification of patients by age group, comorbidity and other variables, which were not possible in our study given our small sample size.

## Conclusions

ADM appears an easy to use prognostic marker for the initial screening of CAP patients referred to a hospital facility. Specifically MR-pro-ADM, is a useful tool for risk stratification of CAP patients, as its levels correlate with indicators linked to the complications of pneumonia, and with outcome. In addition to the initial clinical-laboratory investigations, the peptide may actually be able to provide an early risk stratification in patients affected by pneumonia in term of promptly determining the right level of care.

The present study was a single-center research and contained a relatively small cohort. In order to propose MR-pro-ADM as a routine tool in the Emergency Department well-designed, larger sample and multicenter clinical studies will be needed to further investigate the full potential of our findings.

## Supporting information

S1 Fig MR-pro-ADM levels in relation to age. (DOCX)

S1 Table Comparisons between patients dead and alive at 14 days follow up. (DOCX)

S2 Table Comparisons between dead and alive patients. (DOCX)

S3 Table Comparisons between patients with and without non-invasive mechanical ventilation. (DOCX)

S4 Table Comparisons between patients with and without endotracheal intubation. (DOCX)

S5 Table Comparisons between patients with hospital stay < = 10 day and >10 days. (DOCX)

S6 Table Comparisons between patients with ED stay ≤ 4 days and > 4 days. (DOCX)

S1 File CAPDATABASE. Hospital data of patients with community acquired pneumonia.
(XLS)
