# Supplementary material for: Prognostic performance of MR-pro-adrenomedullin in patients with community acquired pneumonia in the Emergency Department compared to clinical severity scores PSI and CURB
Source: PLoS One. 2017 Nov 21;12(11):e0187702. doi: 10.1371/journal.pone.0187702 (PMC5697810; doi:10.1371/journal.pone.0187702)
Supplement: S1 Table — (DOCX) [file pone.0187702.s002.docx]

**S1 Table. Comparisons between patients dead and alive at 14 days follow up.**

|  | **Whole sample (n=77)** | **Alive (d14) (n=72)** | **Dead (d14)**  **(n=5)** | **p** |
| --- | --- | --- | --- | --- |
| Age | 69.57  +/- 17.43 | 68.54  +/- 17.51 | 84.4  +/- 5.86 | 7e-04 |
| Days of hospitalization in ED | 4.35  +/- 2.96 | 4.32  +/- 2.93 | 4.8 +/- 3.7 | 0.7893 |
| Systolic pressure | 134.27 +/- 23.7 | 134.29 +/- 23.86 | 134 +/- 23.82 | 0.98 |
| Diastolic pressure | 73.96 +/- 13.8 | 73.76 +/- 13.25 | 76.8  +/- 22.11 | 0.7761 |
| Heart rate | 99.94  +/- 21.85 | 99.15  +/- 22.03 | 111.2  +/- 16.83 | 0.1906 |
| Respiratory rate | 20.04  +/- 5.53 | 19.96  +/- 5.45 | 21.2  +/- 7.16 | 0.7216 |
| Oxygen saturation | 91.15 +/- 11.5 | 91.55 +/- 11.77 | 85.4 +/- 3.21 | 0.008 |
| ph | 7.39  +/- 0.11 | 7.4  +/- 0.09 | 7.26 +/- 0.19 | 0.1827 |
| Temperature °C | 37.16 +/- 1.03 | 37.18 +/- 1.04 | 36.84 +/- 0.94 | 0.4777 |
| White cells | 12.24 +/- 5.23 | 12.04 +/- 5.27 | 15.14 +/- 3.94 | 0.1573 |
| Male gender | 47  (61.04 %) | 44  (61.11 %) | 3  (60 %) | 1 |
| Congestive cardiac failure | 32  (41.56 %) | 28  (38.89 %) | 4 (80 %) | 0.182 |
| Kidney failure | 21 (27.27 %) | 19 (26.39 %) | 2 (40 %) | 0.8874 |
| Liver disease | 4 (5.19 %) | 4 (5.56 %) | 0 (0 %) | 1 |
| BPCO | 37 (48.05 %) | 35 (48.61 %) | 2 (40 %) | 1 |
| Tumour | 3 (3.9 %) | 3 (4.17 %) | 0 (0 %) | 1 |
| Diabetes | 12 (15.58 %) | 12 (16.67 %) | 0 (0 %) | 0.7218 |
| Encephalopathy | 23 (29.87 %) | 21 (29.17 %) | 2 (40 %) | 0.9948 |
| Discharge without hospitalization | 19 (24.68 %) | 19 (26.39 %) | 0 (0 %) | 0.4312 |
| Hospitalization | 58 (75.32 %) | 53 (73.61 %) | 5 (100 %) | 0.4312 |
| ICU | 9 (11.69 %) | 7 (9.72 %) | 2 (40 %) | 0.1875 |
| Blood gas | 58 [50 - 75 ] | 59.5 [51.75 - 75.25 ] | 45 [44 - 50 ] | 0.0331 |
| PCR | 83.4 [19.09 - 135.75 ] | 72.05 [16.47 - 132.33 ] | 125 [89.9 - 139.16 ] | 0.1858 |
| MRproADM | 1 [0.55 - 1.76 ] | 0.95 [0.48 - 1.61 ] | 3.68 [2.55 - 5.18 ] | 0.0165 |
| CURB65 | 2 [1 - 2 ] | 1 [1 - 2 ] | 3 [3 - 3 ] | 0.0011 |
| PSI | 4 [2 - 5 ] | 4 [2 - 5 ] | 5 [5 - 5 ] | 0.0171 |
| Kelly | 1 [1 - 2 ] | 1 [1 - 2 ] | 3 [3 - 4 ] | 0.0191 |
