# Supplementary material for: Prognostic performance of MR-pro-adrenomedullin in patients with community acquired pneumonia in the Emergency Department compared to clinical severity scores PSI and CURB
Source: PLoS One. 2017 Nov 21;12(11):e0187702. doi: 10.1371/journal.pone.0187702 (PMC5697810; doi:10.1371/journal.pone.0187702)
Supplement: S2 Table — (DOCX) [file pone.0187702.s003.docx]

**S2 Table. Comparisons between dead and alive patients.**

|  | **Whole sample**  **(n=77)** | **Alive**  **(n=68)** | **Dead**  **(n=9)** | **p** |
| --- | --- | --- | --- | --- |
| Male gender | 47  (61.04 %) | 41  (60.29 %) | 6 (66.67 %) | 0.9962 |
| Congestive heart failure | 32  (41.56 %) | 28  (41.18 %) | 4 (44.44 %) | 1 |
| Kidney failure | 21  (27.27 %) | 18 (26.47 %) | 3 (33.33 %) | 0.9711 |
| Liver disease | 4 (5.19 %) | 4 (5.88 %) | 0 (0 %) | 1 |
| BPCO | 37 (48.05 %) | 31 (45.59 %) | 6 (66.67 %) | 0.404 |
| Tumour | 3 (3.9 %) | 2 (2.94 %) | 1 (11.11 %) | 0.7843 |
| Diabetes | 12 (15.58 %) | 12 (17.65 %) | 0 (0 %) | 0.3774 |
| Encephalopathy | 23 (29.87 %) | 20 (29.41 %) | 3 (33.33 %) | 1 |
| Discharge w/o hospitalization | 19 (24.68 %) | 19 (27.94 %) | 0 (0 %) | 0.1568 |
| Hospitalization | 58 (75.32 %) | 49 (72.06 %) | 9 (100 %) | 0.1568 |
| ICU | 9 (11.69 %) | 5 (7.35 %) | 4 (44.44 %) | 0.0069 |
| Age | 69.57 +/- 17.43 | 68.69 +/- 17.73 | 76.22 +/- 13.96 | 0.1682 |
| Systolic | 134.27 +/- 23.7 | 134.76 +/- 23.68 | 130.56 +/- 24.93 | 0.6424 |
| Diastolic | 73.96 +/- 13.8 | 74.57 +/- 13.06 | 69.33 +/- 18.81 | 0.4387 |
| Heart rate | 99.94 +/- 21.85 | 99.25 +/- 22.31 | 105.11 +/- 18.2 | 0.3957 |
| Respiratory rate | 20.04 +/- 5.53 | 19.76 +/- 5.29 | 22.11 +/- 7.08 | 0.3618 |
| Oxygen saturation | 91.15 +/- 11.5 | 92.38 +/- 10.27 | 81.89 +/- 16.27 | 0.0925 |
| ph | 7.39 +/- 0.11 | 7.41 +/- 0.08 | 7.26 +/- 0.16 | 0.0217 |
| Temperature | 37.16 +/- 1.03 | 37.18 +/- 1.05 | 37.01 +/- 0.93 | 0.6311 |
| White cells | 12.24 +/- 5.23 | 11.95 +/- 5.39 | 14.44 +/- 3.16 | 0.0625 |
| Blood.gas | 58 [50 - 75 ] | 61 [53 - 77.25 ] | 45 [42 - 49 ] | 3e-04 |
| PCR | 83.4 [19.09 - 135.75 ] | 65.59 [15.92 - 132.33 ] | 107 [83.4 - 139.16 ] | 0.1361 |
| MR.proADM | 1 [0.55 - 1.76 ] | 0.9 [0.47 - 1.56 ] | 2.57 [1.61 - 5.18 ] | 0.0011 |
| CURB65 | 2 [1 - 2 ] | 1 [1 - 2 ] | 3 [3 - 3 ] | 8e-04 |
| PSI | 4 [2 - 5 ] | 4 [2 - 4 ] | 5 [5 - 5 ] | 0.0077 |
| Kelly | 1 [1 - 2 ] | 1 [1 - 2 ] | 3 [3 - 4 ] | 0.0027 |
