# Supplementary material for: Prognostic performance of MR-pro-adrenomedullin in patients with community acquired pneumonia in the Emergency Department compared to clinical severity scores PSI and CURB
Source: PLoS One. 2017 Nov 21;12(11):e0187702. doi: 10.1371/journal.pone.0187702 (PMC5697810; doi:10.1371/journal.pone.0187702)
Supplement: S3 Table — (DOCX) [file pone.0187702.s004.docx]

**S3 Table. Comparisons between patients with and without non-invasive mechanical ventilation.**

|  | **Whole sample**  **(n=77)** | **No NIMV**  **(n=61)** | **NIMV**  **(n=16)** | **p** | |
| --- | --- | --- | --- | --- | --- |
| Male Gender | 47 (61.04 %) | 42 (68.85 %) | 5 (31.25 %) | 0.014 |  |
| Congestive cardiac failure | 32 (41.56 %) | 23 (37.7 %) | 9 (56.25 %) | 0.2915 |  |
| Kidney failure | 21 (27.27 %) | 14 (22.95 %) | 7 (43.75 %) | 0.1779 |  |
| Liver disease | 4 (5.19 %) | 3 (4.92 %) | 1 (6.25 %) | 1 |  |
| BPCO | 37 (48.05 %) | 28 (45.9 %) | 9 (56.25 %) | 0.6482 |  |
| Tumor | 3 (3.9 %) | 2 (3.28 %) | 1 (6.25 %) | 1 |  |
| Diabetes | 12 (15.58 %) | 10 (16.39 %) | 2 (12.5 %) | 1 |  |
| Encephalopathy | 23 (29.87 %) | 12 (19.67 %) | 11 (68.75 %) | 4e-04 |  |
| Discharge without hospitalization | 19 (24.68 %) | 19 (31.15 %) | 0 (0 %) | 0.0247 |  |
| Hospitalization | 58 (75.32 %) | 42 (68.85 %) | 16 (100 %) | 0.0247 |  |
| ICU | 9 (11.69 %) | 5 (8.2 %) | 4 (25 %) | 0.1542 |  |
| Age | 69.57 +/- 17.43 | 66.43 +/- 17.82 | 81.56 +/- 8.66 | <0.0001 |  |
| Systolic | 134.27 +/- 23.7 | 135.56 +/- 22.09 | 129.38 +/- 29.38 | 0.4415 |  |
| Diastolic | 73.96 +/- 13.8 | 75.33 +/- 13.84 | 68.75 +/- 12.71 | 0.0827 |  |
| Heart rate | 99.94 +/- 21.85 | 97.69 +/- 20.59 | 108.5 +/- 24.97 | 0.1258 |  |
| Respiratory rate | 20.04 +/- 5.53 | 19.2 +/- 4.95 | 23.25 +/- 6.56 | 0.0321 |  |
| Oxygen saturation | 91.15 +/- 11.5 | 92.93 +/- 8.89 | 84.38 +/- 17.1 | 0.0698 |  |
| ph | 7.39 +/- 0.11 | 7.41 +/- 0.09 | 7.3 +/- 0.12 | 0.0015 |  |
| Temperature | 37.16 +/- 1.03 | 37.27 +/- 1.05 | 36.74 +/- 0.86 | 0.0494 |  |
| White cells | 12.24 +/- 5.23 | 11.38 +/- 3.65 | 15.52 +/- 8.4 | 0.0714 |  |
| Blood gas | 58 [50 - 75 ] | 67 [53 - 76 ] | 45 [41 - 54.5 ] | 2e-04 |  |
| PCR | 83.4 [19.09 - 135.75 ] | 73.9 [16.1 - 130.92 ] | 107.5 [22.92 - 142.12 ] | 0.4073 |  |
| MRproADM | 1 [0.55 - 1.76 ] | 0.89 [0.47 - 1.25 ] | 2.51 [1.59 - 4.15 ] | <0.0001 |  |
| CURB65 | 2 [1 - 2 ] | 1 [1 - 2 ] | 3 [2 - 3 ] | <0.0001 |  |
| PSI | 4 [2 - 5 ] | 3 [1 - 4 ] | 5 [5 - 5 ] | <0.0001 |  |
| Kelly | 1 [1 - 2 ] | 1 [1 - 2 ] | 3 [2.75 - 3.25 ] | <0.0001 |  |
