# Supplementary material for: Prognostic performance of MR-pro-adrenomedullin in patients with community acquired pneumonia in the Emergency Department compared to clinical severity scores PSI and CURB
Source: PLoS One. 2017 Nov 21;12(11):e0187702. doi: 10.1371/journal.pone.0187702 (PMC5697810; doi:10.1371/journal.pone.0187702)
Supplement: S4 Table — (DOCX) [file pone.0187702.s005.docx]

**S4 Table. Comparisons between patients with and without endotracheal intubation.**

|  | **Whole sample**  **(n=77)** | **No ETI**  **(n=71)** | **ETI**  **(n=6)** | **p** |
| --- | --- | --- | --- | --- |
| Male gender | 47 (61.04 %) | 45 (63.38 %) | 2 (33.33 %) | 0.3109 |
| Congestive Heart failure | 32 (41.56 %) | 30 (42.25 %) | 2 (33.33 %) | 1 |
| Kidney failure | 21 (27.27 %) | 20 (28.17 %) | 1 (16.67 %) | 0.8964 |
| Liver disease | 4 (5.19 %) | 4 (5.63 %) | 0 (0 %) | 1 |
| BPCO | 37 (48.05 %) | 33 (46.48 %) | 4 (66.67 %) | 0.5996 |
| Tumor | 3 (3.9 %) | 2 (2.82 %) | 1 (16.67 %) | 0.5586 |
| Diabetes | 12 (15.58 %) | 12 (16.9 %) | 0 (0 %) | 0.6101 |
| Encephalopathy | 23 (29.87 %) | 22 (30.99 %) | 1 (16.67 %) | 0.7861 |
| Discharge without hospitalization | 19 (24.68 %) | 19 (26.76 %) | 0 (0 %) | 0.3336 |
| Hospitalization | 58 (75.32 %) | 52 (73.24 %) | 6 (100 %) | 0.3336 |
| ICU | 9 (11.69 %) | 4 (5.63 %) | 5 (83.33 %) | <0.0001 |
| Age | 69.57 +/- 17.43 | 69.49 +/- 17.91 | 70.5 +/- 11.18 | 0.8469 |
| Systolic | 134.27 +/- 23.7 | 133.72 +/- 23.31 | 140.83 +/- 29.57 | 0.5881 |
| Diastolic | 73.96 +/- 13.8 | 73.25 +/- 13.22 | 82.33 +/- 18.83 | 0.2956 |
| Heart rate | 99.94 +/- 21.85 | 98.72 +/- 22.04 | 114.33 +/- 13.47 | 0.0353 |
| Respiratory rate | 20.04 +/- 5.53 | 20.1 +/- 5.46 | 19.33 +/- 6.74 | 0.7965 |
| Oxygen saturation | 91.15 +/- 11.5 | 92.08 +/- 10.15 | 80.17 +/- 20.27 | 0.2113 |
| ph | 7.39 +/- 0.11 | 7.41 +/- 0.08 | 7.18 +/- 0.11 | 0.0042 |
| Temperature | 37.16 +/- 1.03 | 37.17 +/- 1.03 | 36.98 +/- 1.02 | 0.6797 |
| White cells | 12.24 +/- 5.23 | 11.88 +/- 5.18 | 16.43 +/- 4.05 | 0.0394 |
| Blood gas | 58 [50 - 75 ] | 60 [51.5 - 75.5 ] | 45.5 [42 - 52 ] | 0.0117 |
| PCR | 83.4 [19.09 - 135.75 ] | 73.9 [17.84 - 133.47 ] | 86.65 [79.35 - 126.72 ] | 0.4939 |
| MRproADM | 1 [0.55 - 1.76 ] | 0.92 [0.52 - 1.58 ] | 3.12 [2.5 - 4.8 ] | 0.018 |
| CURB65 | 2 [1 - 2 ] | 1 [1 - 2 ] | 2.5 [1.25 - 3 ] | 0.2073 |
| PSI | 4 [2 - 5 ] | 4 [2 - 5 ] | 5 [4.25 - 5 ] | 0.0231 |
| Kelly | 1 [1 - 2 ] | 1 [1 - 2 ] | 3 [3 - 3.75 ] | 0.001 |

ETI: endotracheal intubation.
