# Supplementary material for: Prognostic performance of MR-pro-adrenomedullin in patients with community acquired pneumonia in the Emergency Department compared to clinical severity scores PSI and CURB
Source: PLoS One. 2017 Nov 21;12(11):e0187702. doi: 10.1371/journal.pone.0187702 (PMC5697810; doi:10.1371/journal.pone.0187702)
Supplement: S5 Table — (DOCX) [file pone.0187702.s006.docx]

**S5 Table. Comparisons between patients with hospital stay <= 10 day and >10 days.**

|  | **Whole sample**  **(n=77)** | **Hospital stay <= 10 days (n=39(** | **Hospital stay > 10 days**  **(n=38)** | **p** |
| --- | --- | --- | --- | --- |
| Male Gender | 47 (61.04 %) | 26 (66.67 %) | 21 (55.26 %) | 0.4283 |
| Congestive Cardiac failure | 32 (41.56 %) | 14 (35.9 %) | 18 (47.37 %) | 0.4296 |
| Kidney failure | 21 (27.27 %) | 9 (23.08 %) | 12 (31.58 %) | 0.5608 |
| Liver disease | 4 (5.19 %) | 2 (5.13 %) | 2 (5.26 %) | 1 |
| BPCO | 37 (48.05 %) | 14 (35.9 %) | 23 (60.53 %) | 0.053 |
| Tumor | 3 (3.9 %) | 0 (0 %) | 3 (7.89 %) | 0.2298 |
| Diabetes | 12 (15.58 %) | 5 (12.82 %) | 7 (18.42 %) | 0.7165 |
| Encephalopathy | 23 (29.87 %) | 8 (20.51 %) | 15 (39.47 %) | 0.1168 |
| Discharge w/o hospitalization | 19 (24.68 %) | 18 (46.15 %) | 1 (2.63 %) | <0.0001 |
| Hospitalization | 58 (75.32 %) | 21 (53.85 %) | 37 (97.37 %) | <0.0001 |
| ICU | 9 (11.69 %) | 2 (5.13 %) | 7 (18.42 %) | 0.1442 |
| Age | 69.57 +/- 17.43 | 65.18 +/- 19.53 | 74.08 +/- 13.82 | 0.0237 |
| Systolic | 134.27 +/- 23.7 | 133.92 +/- 24.93 | 134.63 +/- 22.7 | 0.8966 |
| Diastolic | 73.96 +/- 13.8 | 74.49 +/- 13.69 | 73.42 +/- 14.07 | 0.7372 |
| Heart rate | 99.94 +/- 21.85 | 98.41 +/- 19.89 | 101.5 +/- 23.85 | 0.5395 |
| Respiratory rate | 20.04 +/- 5.53 | 19.41 +/- 5.53 | 20.68 +/- 5.52 | 0.315 |
| Oxygen saturation | 91.15 +/- 11.5 | 94.24 +/- 3.96 | 87.98 +/- 15.34 | 0.0192 |
| ph | 7.39 +/- 0.11 | 7.42 +/- 0.07 | 7.36 +/- 0.13 | 0.0198 |
| Temperature | 37.16 +/- 1.03 | 37.65 +/- 1 | 36.66 +/- 0.79 | <0.0001 |
| White cells | 12.24 +/- 5.23 | 11.39 +/- 3.69 | 13.11 +/- 6.37 | 0.156 |
| Blood.gas | 58 [50 - 75 ] | 69 [54.5 - 81 ] | 55.5 [46.25 - 67 ] | 0.0034 |
| PCR | 83.4 [19.09 - 135.75 ] | 89.9 [26.77 - 136.96 ] | 69.5 [16.22 - 134.61 ] | 0.4852 |
| MRproADM | 1 [0.55 - 1.76 ] | 0.74 [0.46 - 1.19 ] | 1.55 [0.86 - 2.46 ] | 0.0028 |
| CUR.65 | 2 [1 - 2 ] | 1 [1 - 2 ] | 2 [1 - 3 ] | 0.0571 |
| PSI | 4 [2 - 5 ] | 3 [1 - 4 ] | 4 [3.25 - 5 ] | 0.0024 |
| Kelly | 1 [1 - 2 ] | 1 [1 - 2 ] | 2 [1 - 3 ] | 0.0887 |
