# Supplementary material for: Prognostic performance of MR-pro-adrenomedullin in patients with community acquired pneumonia in the Emergency Department compared to clinical severity scores PSI and CURB
Source: PLoS One. 2017 Nov 21;12(11):e0187702. doi: 10.1371/journal.pone.0187702 (PMC5697810; doi:10.1371/journal.pone.0187702)
Supplement: S6 Table — (DOCX) [file pone.0187702.s007.docx]

**S6 Table. Comparisons between patients with ED stay ≤ 4 days and > 4 days.**

|  | **Whole sample**  **(n=77)** | **ED stay <= 4 days**  **(n=43)** | **ED stay > 4 days**  **(n=34)** | **p** |
| --- | --- | --- | --- | --- |
| Male Gender | 47 (61.04 %) | 26 (60.47 %) | 21 (61.76 %) | 1 |
| Congestive cardiac failure | 32 (41.56 %) | 23 (53.49 %) | 9 (26.47 %) | 0.0311 |
| Kidney failure | 21 (27.27 %) | 10 (23.26 %) | 11 (32.35 %) | 0.5271 |
| Liver disease | 4 (5.19 %) | 3 (6.98 %) | 1 (2.94 %) | 0.7831 |
| BPCO | 37 (48.05 %) | 18 (41.86 %) | 19 (55.88 %) | 0.3206 |
| Tumor | 3 (3.9 %) | 2 (4.65 %) | 1 (2.94 %) | 1 |
| Diabetes | 12 (15.58 %) | 8 (18.6 %) | 4 (11.76 %) | 0.6133 |
| Encephalopathy | 23 (29.87 %) | 10 (23.26 %) | 13 (38.24 %) | 0.2398 |
| Discharge without hospitalization | 19 (24.68 %) | 6 (13.95 %) | 13 (38.24 %) | 0.0287 |
| Hospitalization | 58 (75.32 %) | 37 (86.05 %) | 21 (61.76 %) | 0.0287 |
| ICU | 9 (11.69 %) | 3 (6.98 %) | 6 (17.65 %) | 0.2757 |
| Age | 69.57 +/- 17.43 | 66.84 +/- 18.36 | 73.03 +/- 15.76 | 0.1158 |
| Systolic pressure | 134.27 +/- 23.7 | 132.21 +/- 21.05 | 136.88 +/- 26.78 | 0.4075 |
| Diastolic pressure | 73.96 +/- 13.8 | 72.23 +/- 12.2 | 76.15 +/- 15.5 | 0.2322 |
| Heart rate | 99.94 +/- 21.85 | 100.26 +/- 21.08 | 99.53 +/- 23.09 | 0.8872 |
| Respiratory rate | 20.04 +/- 5.53 | 20.07 +/- 5.64 | 20 +/- 5.47 | 0.9564 |
| Oxygen saturation | 91.15 +/- 11.5 | 93.77 +/- 6.52 | 87.84 +/- 15.18 | 0.0391 |
| ph | 7.39 +/- 0.11 | 7.39 +/- 0.09 | 7.38 +/- 0.12 | 0.5925 |
| Temperature | 37.16 +/- 1.03 | 37.18 +/- 1.02 | 37.12 +/- 1.06 | 0.8015 |
| White cells | 12.24 +/- 5.23 | 11.93 +/- 3.92 | 12.63 +/- 6.57 | 0.5804 |
| Blood gas | 58 [50 - 75 ] | 67 [55 - 85.5 ] | 54 [49 - 71.25 ] | 0.0273 |
| CRP | 83.4 [19.09 - 135.75 ] | 73.9 [14.2 - 128.58 ] | 86.65 [23.78 - 150.75 ] | 0.3832 |
| MRproADM | 1 [0.55 - 1.76 ] | 0.88 [0.42 - 1.56 ] | 1.23 [0.86 - 1.93 ] | 0.0433 |
| CURB65 | 2 [1 - 2 ] | 1 [1 - 2 ] | 2 [1 - 2.75 ] | 0.1769 |
| PSI | 4 [2 - 5 ] | 4 [1 - 5 ] | 4 [3 - 5 ] | 0.2957 |
| Kelly | 1 [1 - 2 ] | 1 [1 - 2 ] | 2 [1 - 3 ] | 0.0342 |
