# Supplementary material for: Prognostic performance of MR-pro-adrenomedullin in patients with community acquired pneumonia in the Emergency Department compared to clinical severity scores PSI and CURB
Source: PLoS One. 2017 Nov 21;12(11):e0187702. doi: 10.1371/journal.pone.0187702 (PMC5697810; doi:10.1371/journal.pone.0187702)
Supplement: Supplementary file 7 [file pone.0187702.s001.docx]

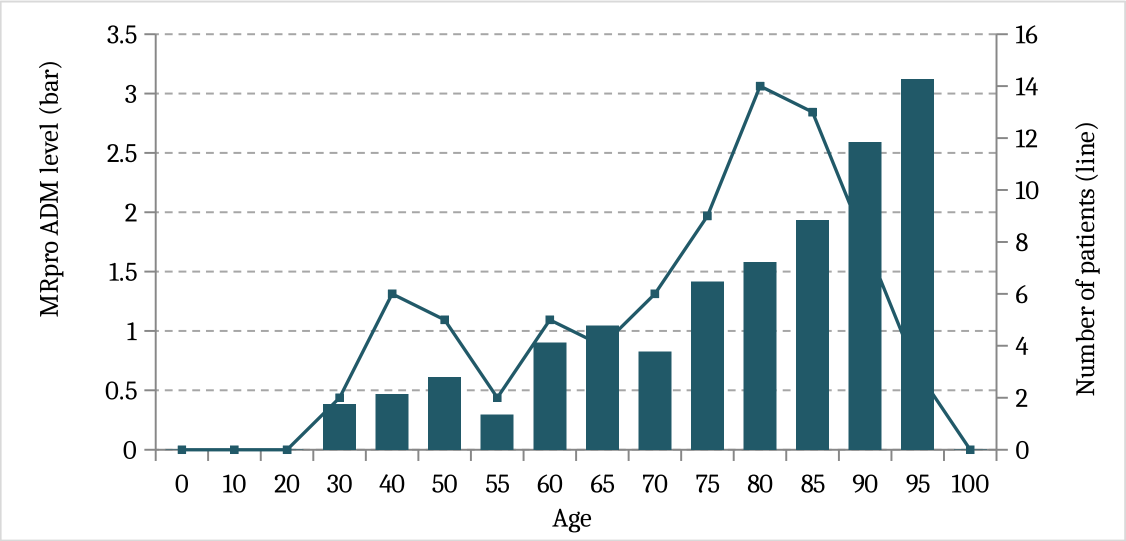

MRpro ADM level (bar)
3.5
3
2.5
2
1.5
1
0.5
0
16
14
12
10
8
6
4
2
0
Number of patients (line)
0 10 20 30 40 50 55 60 65 70 75 80 85 90 95 100
Age
